# Exploration of a rare population of Chinese chestnut in North America: stand dynamics, health and genetic relationships

**DOI:** 10.1093/aobpla/plu065

**Published:** 2014-10-20

**Authors:** Amy C. Miller, Keith E. Woeste, Sandra L. Anagnostakis, Douglass F. Jacobs

**Affiliations:** 1 Department of Forestry and Natural Resources, Hardwood Tree Improvement and Regeneration Center, Purdue University, 715 West State St., West Lafayette, IN 47907, USA; 2 USDA Forest Service, Northern Research Station, Hardwood Tree Improvement and Regeneration Center, 715 West State St., West Lafayette, IN 47907, USA; 3 The Connecticut Agricultural Experiment Station, New Haven, CT 06504, USA

**Keywords:** Dendrochronology, distribution, ecology, hybrid, invasive, root DNA barcoding.

## Abstract

With the transport of plants around the globe, exotic species can readily spread disease to their native relatives; however, they can also provide genetic resistance to those relatives through hybrid breeding programmes. American chestnut (*Castanea dentata*) was an abundant tree species in North America until its decimation by introduced chestnut blight. To restore chestnut in North America, efforts are ongoing to test putative blight-resistant hybrids of *Castanea dentata* and Chinese chestnut (*Castanea mollissima*), but little is known about the ecology of *C. mollissima*. In a forest in northeastern USA in which *C. mollissima* has become established, we explored questions of stand dynamics, health and genetic relationships of *C. mollissima* offspring to an adjacent parent orchard. We found that *C. mollissima* was adapted and randomly distributed among native species in this relatively young forest. The genetics of the *C. mollissima* population compared with its parents indicated little effect of selection pressure as each of the parent trees contributed at least one offspring. The ease with which this exotic species proliferated calls to question why *C. mollissima* is rare elsewhere in forests of North America. It is likely that a time window of low animal predation allowed seedlings to establish, and the shallow soil at this site limited the maximum forest canopy height, permitting the characteristically short-statured *C. mollissima* to avoid suppression. Our results indicate that because *C. mollissima* exhibited pioneer species characteristics, hybrids between *C. mollissima* and *C. dentata* have the potential to be successful pioneer species of future forests in North America, and we challenge the paradigm that exotic tree species are wholly detrimental to native biodiversity. We contend that exotic tree species should be assessed not only by their level of threat to native species, but also by their potential positive impacts on ecosystems via hybrid breeding programmes.

## Abstract (precis)

With worldwide transport of plants, exotic species spread disease to native relatives, but they can also provide disease resistance via hybrid breeding programs. American chestnut was nearly eliminated from North America by introduced chestnut blight, but resistant hybrids of American and Chinese chestnuts are being created. We studied the ecology and genetics of an unmanaged population of Chinese chestnut in North America. The forest dynamics of pure Chinese chestnut indicate that hybrids between the Chinese and American species could be successful colonizers of forests in North America. We challenge the paradigm that exotic tree species are wholly detrimental to native biodiversity.

## Introduction

Anthropogenic activities increasingly influence natural systems worldwide; an example is the movement of plant species and their diseases around the globe. Land managers have a decreasing ability to keep ‘native’ and ‘exotic’ plants separate, especially when dealing with species of economic value, and as a consequence diseases have jumped from co-evolutionary plant hosts to their more susceptible relatives in other parts of the world. Although exotic species may harbour diseases, they also possess the genetic potential to resist those diseases, and tree breeders in the USA strive to capture those resistance genes to restore threatened native species (Michler *et al.* 2006; Merkle *et al.* 2007; Jacobs *et al.* 2013).

Chestnuts (genus *Castanea*, family Fagaceae) occur in temperate climates worldwide, and have ecological, economic and cultural importance in their native ranges of Europe, the Caucasus Mountains and eastern Asia (Conedera *et al.* 2004; Davis 2006). Historically, *Castanea dentata* (American chestnut) was also of great importance in eastern North America, until it was nearly extirpated from its native range in the early 20th century by chestnut blight disease, caused by the introduced fungus *Cryphonectria parasitica* (Anagnostakis 2012). The fungus was accidentally introduced from eastern Asia with early importation of *Castanea crenata* (Japanese chestnut) for commercial purposes (Powell 1898; Shear and Stevens 1916). Imported slightly later, *Castanea mollissima* (Chinese chestnut) has now been grown in cultivation in the USA for over a century. Its broad adaptability to soil and climatic conditions in eastern North America has made it commercially more valuable for nut production than the Japanese species (Hunt *et al.* 2009; Fei *et al.* 2012); however, there is still a desire to recapture the economical and ecological value of *C. dentata* as a forest tree and timber species. Current chestnut breeding programmes aimed at re-establishing American chestnut via hybridization of *C. dentata* and *C. mollissima* (and other *Castanea*), which are being carried out by organizations such as The American Chestnut Foundation and the Connecticut Agricultural Experiment Station (Burnham *et al.* 1986; Hebard 2001; Anagnostakis 2012). These breeding programmes aim to transfer resistance to chestnut blight from the genome of Asian *Castanea* species into Asian × American chestnut hybrids (Jacobs 2007; Anagnostakis 2012). One concern with hybrids is that although *C. mollissima* is a canopy co-dominant in forests of northern China (Steiner *et al.* 2009), the genetic stock available to breeding programmes in the USA seems to be limited in height, possibly due to its selection for nut production over timber form (Fei *et al.* 2012).

Ideally, hybrid *Castanea* will be able to establish and reproduce in habitats formerly occupied by *C. dentata* without becoming overly aggressive. There is some concern that breeding programmes will result in *Castanea* hybrids that are weedy and invasive and will displace native hardwoods (Jacobs 2007; Jacobs *et al.* 2013). Unlike Asian woody plants such as *Lonicera* spp., *Ailanthis altissima* and *Elaeagnus* spp., however, non-native *Castanea*, including *C. mollissima*, have not shown strong invasive tendencies in North America (Sakai *et al.* 2001; Zheng *et al.* 2004). There are few cases of *C. mollissima* escaping its orchard bounds to become established in the forest among native North American species. Test plantings of *C. mollissima*, *C. dentata* and *Castanea* hybrids made by the United States Department of Agriculture (USDA) in 1947–55 (Diller *et al.* 1964) were relocated and evaluated (Berry 1980; Schlarbaum *et al.* 1994), and only a few have persisted to date.

If *Castanea* hybrids are to survive and proliferate in North American forests, they must be adapted to North American forest conditions; however, little is known about the ecology, silvics and genetics of *C. mollissima*. There is only one case of naturalized *C. mollissima* in the USA reported in the scientific literature (Jaynes 1965; Anagnostakis 1992): a forest plot of ∼1 ha on a private property near Dayville, CT, USA. We conducted studies in this forest and in an adjacent orchard grove of ∼85-year-old *C. mollissima* trees, the ‘parent orchard.’ *Castanea mollissima* in this disjunct USA stand seem to be well integrated into a natural forest among native North American species.

The objectives of this study were to understand the factors that permitted *C. mollissima* to establish in a second-growth mixed forest in CT and, more generally, to understand if *C. mollissima* has the potential to persist in North American forests. The overarching goal of our research is to surmise, through observations and synthesis of the literature, the potential for *Castanea* hybrids to survive and compete effectively in future North American forests. This instance of *C. mollissima* infiltrating unmanaged land in CT is the only documented case of that species co-dominating a North American forest; therefore, we gained rare insight into its performance in an unmanaged setting. We examined forest composition, spatial distributions of woody stems, horizontal and vertical distributions of woody roots, stand dynamics and vigour of *C. mollissima*, and genetic relationships among all *C. mollissima* individuals. These analyses allowed us to explore spatial partitioning and competition above and below ground, disturbance history of the forest, associations of site quality and health of *C. mollissima* and the possibility of a genetic bottleneck between *C. mollissima* orchard parents and forest offspring.

## Methods

### Field site

The Dayville woodlot is a forest of ∼1 ha near Dayville, CT, USA (41–50′47″N, 071–53′15″W). The woodlot and a *C. mollissima* orchard immediately adjacent were visited in May 2012. The parent orchard sits on level ground atop a hill, and the woodlot is on a naturally terraced hillside with the slope ranging from 0 to ∼25° (0–47 %). Jaynes (1965) characterized the soil of the Dayville woodlot as sandy loam, with pH ranging from 4.4 to 4.8. At the time the parent orchard was planted in 1926, the woodlot was a grazed agricultural field that was subsequently abandoned and left to naturally regenerate into forest. The parent orchard, originally planted with 67 trees, was assessed for survival in 2012, and 28 trees were found, probably corresponding to the 28 found by Anagnostakis in 1992. Each parent tree was given a unique identification number preceded by a ‘P’ (e.g. P1).

The seeds from which the orchard trees grew were brought to the USA by the USDA in 1925 under the label of Plant Introduction (PI) 58602 (Anagnostakis 1992). While the seeds were officially imported from Nanking, in southern China, seeds were requested from other parts of the country, and especially the north, so that they might be more climatically adapted to the northern USA. Plant Introduction 58602 included seed collections from many small lots in China, though no records were kept of their exact origins (Anagnostakis 1992). It was subsequently dispersed widely across the USA (i.e. 7826 seedlings were sent out to 85 locations). Therefore, to determine if the survival and growth of *C. mollissima* in the Dayville woodlot was the result of a common genetic background, we assessed genetic relatedness within the orchard parent population, within the forest offspring population and between these populations.

### Forest structure

We conducted several forest-wide analyses of the Dayville woodlot to detect characteristics that might explain the invasion and persistence of *C. mollissima* at that location. To assess the sizes and distributions of living woody stems, all trees and shrubs >5 cm diameter-at-breast-height (DBH) in the forest were mapped by polar coordinates using a range finder and sighting compass, and the coordinates were used to create a ‘master map.’ All *C. mollissima* in the forest (hereafter ‘offspring’) were systematically given a unique identification number (1–72). For all trees, we recorded species identification and DBH, grouping them into 5-cm size classes. Anecdotal observations such as presence of woody vines, understorey composition and evidence of seedling regeneration were noted to aid the interpretation of our results.

### Root DNA

**Figure 1.**
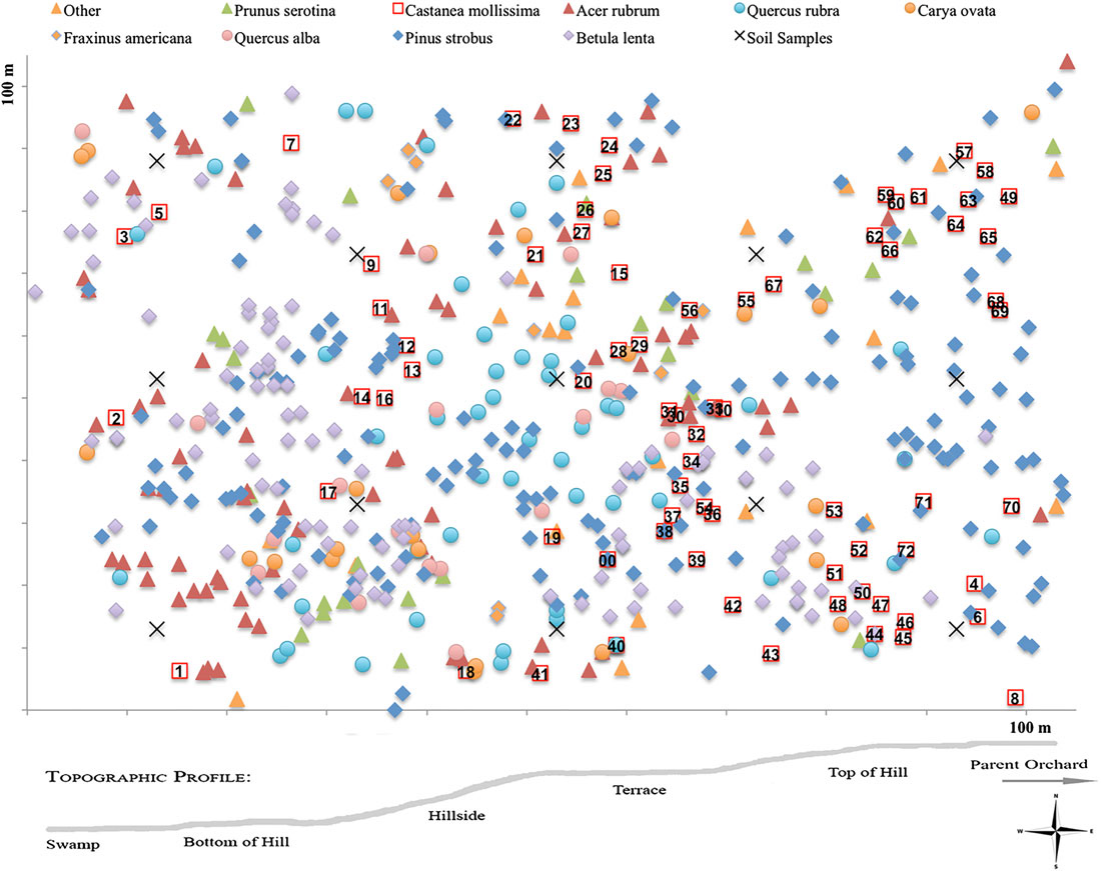
Map of tree species in the naturalized stand of *C. mollissima* in CT, USA. Chestnuts are individually labelled by the unique identification number. Forest is 100 × 100 m. Topographic profile reveals microsites identified in the study.

Because forest establishment and individual tree growth are heavily influenced by soil qualities, root dynamics and below-ground competition, we analysed the distributions of woody roots at the study site. As with the above-ground forest structure, we were looking for below-ground explanations for the success of *C. mollissima* in the forest. Soil cores were taken from 13 locations at regularly spaced intervals throughout the forest (Fig. 1) using a 6.25-cm diameter soil probe. At each location, soil samples were taken at 0–15, 15–30, 30–45 and 45–60 cm, or until reaching bedrock. The most common depth to bedrock was 30 cm. In total, 25 soil cores were taken. Fine roots were removed from the soil by hand and dried in a desiccator using a Dri-Rite desiccant (Chicago, IL). Twelve fine root fragments were selected at random from each core for DNA extraction. Roots were placed in a wash solution of 0.01 % Triton X-100 in Tris–HCl and tumbled in a rotisserie at room temperature for 1 day to remove as much dirt and other contaminants as possible from the root surface. Roots were rinsed in nanopure water, and DNA extraction proceeded as described under relatedness and parentage (below).

To determine the species associated with root samples removed from soil cores, the *rbcL* genes from DNA of pooled root samples were sequenced using Illumina MiSeq (Illumina, Inc., San Diego, CA) run with *rbcL* PCR primers from Kress *et al.* (2009) and Illumina step-out PCR primers from the Illumina Customer Sequence Letter (2012). All DNA sequences were grouped by soil core (depth and location). Raw sequences were processed into FastA files to determine the number of reads and the number of bases per read. SAMtools (flagstat and idxstats) software was used to translate reads into BAM files for further processing and to confirm the percentage of properly paired and singleton reads and the percentage of mapped and unmapped reads (Li *et al.* 2009). Fastx_clipper was used to remove adapters and poor quality bases on both the 5′ and 3′ ends. Small reads (<30 bases) were discarded. Root *rbcL* sequences were cross-referenced against the GenBank^®^ database to determine the best set of reference sequences for woody species present in the Dayville woodlot. Resolution to genus was achieved with 642 reference sequences. Read-to-reference mapping was performed using Bowtie2. Both global and local alignments were made, but because of their higher sensitivity and accuracy, global alignments were chosen for further analysis.

### Tree health and vigour

We examined individual *C. mollissima* offspring trees to account for characteristics, history and environmental relationships that might help to elucidate the species' success in this forest. For each offspring we recorded canopy position (dominant, co-dominant, intermediate or suppressed), a rank of chestnut blight canker severity (using a system of 0–3 capturing the range of severity within that population based on the amount, size and level of healing of cankers) and the presence of epicormic branches, which indicate a decline in tree health. We distinguished six distinct microsites in the forest characterized by their slope position and proximity to the forest edge. The microsite of each offspring tree was recorded.

### Dendrochronology

Twenty-five offspring of *C. mollissima* were chosen at random from among the population of 72, and two increment cores were taken at breast height from each. Cores were taken parallel to the slope of the hill to avoid tension wood. Cores were dried and sanded, and raw ring widths were measured using the Velmex measuring stage and Measure J2X tree ring software. Skeleton plots were used to align dates of the cores, and dated measurement files were checked for accuracy with the program COFECHA (Grissino-Mayer 2001).

### Relatedness and parentage

We estimated genetic relatedness and parentage of the offspring to determine if the parent trees (in the orchard) contributed unequally to the offspring cohort. Leaves were collected for DNA extraction from each of the 72 offspring and the 28 parent trees in the orchard. A slingshot was used to sample leaves because of the canopy height (∼18 m) of the forest and orchard trees. The leaves were placed in 50 mL conical tubes and kept in a cooler with ice and subsequently in a freezer at −80°C. They were taken immediately from the freezer and freeze-dried for long-term storage. DNA was extracted in a buffer of cetyltrimethyl ammonium bromide and 2-mercaptoethanol and purified by phenol–chloroform extraction (Doyle and Doyle 1987; Zhao and Woeste 2011). Sodium chloride and cold isopropanol were used to precipitate the DNA, which was then dried and re-suspended in TE buffer. A nanodrop 8000 spectrophotometer (Thermo Scientific, Wilmington, DE) was used to quantify the DNA, which was diluted to ∼50 ng µL^−1^ with nanopure water. Eleven simple sequence repeat (SSR) markers (Table 2) were used for genotyping (Inoue *et al.* 2009; Kubisiak *et al.* 2013). To increase flexibility in post-PCR multiplexing, an M13 sequence (5′-AGTAAAACGACGGCCAGT-3′) was added to the 5′ end of each forward primer (Schuelke 2000), and three-primer PCR was performed including a dye (HEX or FAM) labelled with the same M13 sequence. The PCR thermal profile was a 4-min denaturation at 95 °C, 20 cycles of 30 s at 94 °C, 30 s at 65 °C and 1 min at 72 °C, with the annealing temperature stepping down 0.5 °C every cycle (from 65 °C to 55 °C), followed by 20 cycles of the same, with a constant annealing temperature of 55 °C. The final extension was 72 °C for 5 min. Genemapper (Applied BioSystems) was used to score the genotypes.

### Data analyses

Forest structure data were used to determine the number of stems per species, to create graphs of size-class distributions and to determine the percent basal area (BA) of each species. The polar location coordinates were translated into Euclidean coordinates, which were then used for Ripley's *K* analysis of spatial distribution in the statistical platform R to determine the spatial pattern of all trees in the forest. Ripley's Cross *K* was used to establish baseline forest characteristics, determining if there was spatial clustering or repulsion between *C. mollissima* and all other trees and between shade-tolerant and shade-intolerant tree species. Because field observations suggested that *C. mollissima* and *Pinus strobus* were clustered together, we used Ripley's Cross K to test those spatial relationships. Ripley's *K* and Cross-*K* statistics are indices to examine whether spatial point patterns are clustered, random or uniform. They combine methods of nearest neighbour counts and quadrat surveys into a second-order statistic that looks at the variances of distances to neighbours in two-dimensional space using concentric circles with increasing radii from each sample point (Haase 1995). For both analyses, an isotopic edge correction was used to eliminate the false empty space outside the edge of plot boundaries. Ripley's *K* values were converted to *L*-values [*L* = (*K*/π)^1/2^] for clear graphic representation. Chi-squared tests of association (*α* = 0.05) were used to evaluate the significance of associations between microsites, canker severity, canopy position and the presence of epicormic branches for *C. mollissima*.

The number of root sequence reads per genus was used as a proxy for root abundance in each soil core/location. Inverse modelling was used to fit functions for the distribution of roots relative to the distribution of above-ground stems, much like the techniques used to model seed dispersal from seed trap data (Nathan and Muller-Landau 2000; Jones *et al.* 2011). Contour-style maps were generated from the model showing the varying abundance of roots per genus across the forest. Because of orders of magnitude of difference in numbers of reads/genus/location, a log transformation of read number was applied for further analysis. Root abundance per genus was pooled across each sampling depth (15-cm increment) to show changes in abundance of all genera from the surface to 60 cm. Root abundance of *C. mollissima* within each microsite was compared with BA of the *C. mollissima* stems per microsite to determine if site conditions, root abundance and stem size were related.

Ring widths from the two cores from each tree were counted to determine the approximate ages/number of cohorts and averaged to analyse the growth history of each cohort. Tree-ring measurement files were processed with programmes FMT, YUX, CASE and finally JOLTS (http://web.utk.edu/~grissino/). JOLTS detects suppression and release events by calculating a moving average for a specified time window and specified sensitivity. For release events, a 10-year time window was used, based on the strategy of Nowacki and Abrams (1997), and the data were run using three sensitivity levels (minimum release factors): 10, 50 and 100 % growth change of raw ring widths. For suppression events, a 10-year time window was used with a minimum suppression factor of 100 %. Additionally, raw ring-width measurements were detrended for ontogenetic growth decline and compiled into a Master chronology using the program CRONOL (http://web.utk.edu/~grissino/). The National Oceanic and Atmospheric Administration's National Climate Data Center was used to gather precipitation data and cooling degree days in CT spanning the same years as the Master chronology (http://www.ncdc.noaa.gov/cag/time-series). Cooling degree days, calculated by summing each day's average temperature minus 18 °C, are proxies for heat and light and were used to compare relative amounts of heat and light throughout the years. Simple linear regression was used to test for correlations between stand-wide increment growth and each year's annual precipitation, each year's growing season precipitation, the previous autumn's precipitation and the previous 18 months of precipitation. Multiple linear regression was used to determine correlations between growth and precipitation and heat/light intensity per year.

Genetic diversity and relatedness were calculated using GenAlEx (Peakall and Smouse 2012). Pairwise relatedness, heterozygosity, *F*_st_, *F*_is_, *F*_it_, private alleles and a genetic distance matrix were calculated between the two populations (parent and offspring), and tests for Hardy–Weinberg equilibrium were conducted for each locus. For pairwise relatedness, the Lynch and Ritland (1999) method was used. The genetic distance matrix was used to run a principal component analysis (PCA) based on correlations and SSR frequencies. For parentage analysis, the software CERVUS (Kalinowski *et al.* 2007) was used. Simulations were run for parent pairs of 10 000 simulated offspring, parent sexes unknown, with a 92 % genotype success rate. The proportion of candidate parents sampled was set at 0.6 because Jaynes found 35 reproducing orchard trees in 1965, yet for this study only 28 were remaining. A relaxed confidence (80 %) was used to assign parents. For offspring to which no parent pair could be confidently assigned, non-excluded parents were recorded, meaning those putative parents that had no loci mismatching. Randomness of the parent contribution to the offspring population was tested by bootstrapping with 95 % confidence.

## Results

### Forest structure

**Figure 2.**
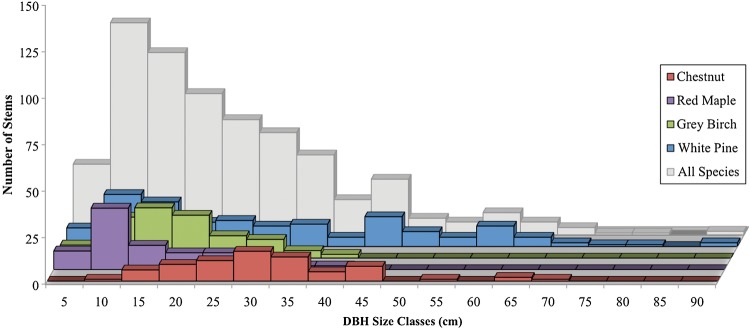
Size-class distributions of all species in the Dayville woodlot (grey) with the four most numerous species shown individually.

Common woody species (more than eight individuals) in the Dayville woodlot included *P. strobus*, *C. mollissima*, *Betula lenta*, *Acer rubrum*, *Quercus rubra*, *Quercus alba*, *Carya ovata*, *Prunus serotina* and *Fraxinus americana* (Figs 1 and 2, Table 1). Other species (less than eight individuals) included *Populus grandidentata*, *Juniperus virginiana*, *Ulmus americana*, *Malus* *× domestica*, *Cornus alternifolia*, *Cornus florida*, *Euonymus alatus*, *Lonicera maackii* and *Sassafras albidum*. Stem abundance and BA were determined for the most common species (Table 1). *Pinus strobus* was the most abundant species with 168 stems that accounted for 50 % of BA. *Castanea mollissima* was the fourth most abundant species with 72 stems, but it ranked second in BA, accounting for 18 %. *Betula lenta* was the second most abundant (108 stems), but its smaller stems accounted for only 8 % of the BA. Similarly, *A. rubrum* was the third most abundant species (80 stems) but accounted for only 5 % of BA. The other noteworthy species was *Q. rubra*, which comprised 50 stems and 9 % of BA.

**Table 1.** Species, stem densities and BA in the Dayville woodlot. *P. grandidentata, *J. virginiana*, *U. americana*, *Malus × domestica*, *C. alternifolia*, *C. florida*, *E. alatus*, *L. maackii*, *S. albidum*.

Species	Stems (ha^−1^)	BA (m^2^ ha^−1^)	BA (%)
*P. strobus*	168	15.07	50
*C. mollissima*	72	5.51	18
*Q. rubra*	50	2.84	9
*B. lenta*	108	2.55	8
*A. rubrum*	80	1.39	5
*P. serotina*	27	0.96	3
*C. ovate*	24	0.33	1
*F. americana*	8	0.32	1
*Q. alba*	18	0.28	1
Others*	23	1.12	4

Size-class distributions (Fig. 2) of the various species showed that *P. strobus* occupied the entire range of size classes, monotonically decreasing from 10 to 90 cm DBH. *Castanea mollissima* had a bell-shaped size distribution with peak abundance at 30 cm DBH. *Pinus strobus* and *C. mollissima* were the only species to have stems >50 cm DBH. *Acer rubrum* and *B. lenta* had bell-shaped distributions with peaks at 10 and 15 cm, respectively. *Quercus rubra* had fairly even size distributions from 10 to 40 cm.

### Spatial distribution

**Figure 3.**
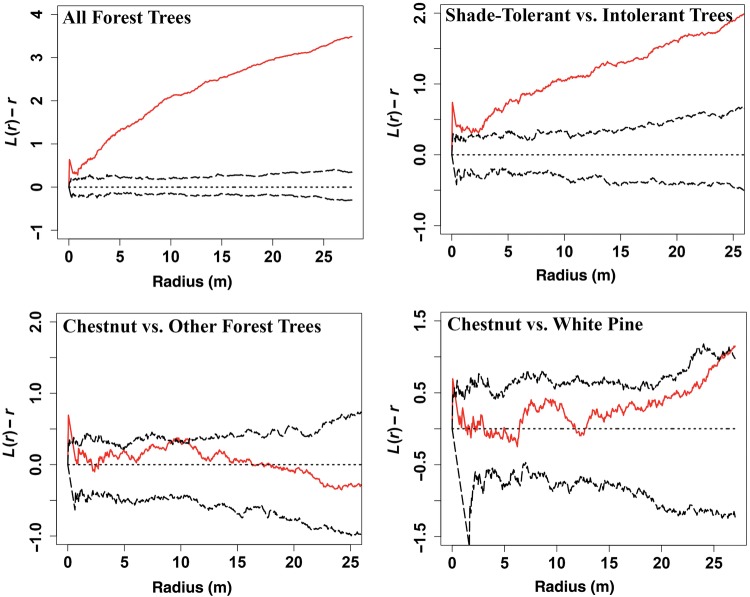
Ripley's *K* and Cross-*K* (converted to *L*-values) indicating patterns of spatial distribution. All forest trees are clustered. Shade-tolerant trees cluster around shade-intolerant trees. Chestnut is randomly distributed among all forest trees. Chestnut does not significantly cluster with white pine.

Ripley's *K* analysis (Fig. 3) indicated that all forest trees were clustered. Ripley's Cross-*K* analysis showed that shade-tolerant trees clustered around shade-intolerant trees, which is consistent with expectations of a naturally generated, unmanaged forest. Ripley's Cross-*K* analysis of *C. mollissima* showed that it was randomly distributed among all other forest trees and randomly distributed with respect to *P. strobus*, though the two species showed a trend towards clustering with each other. These two species were the largest in the forest, and the most likely pioneer colonizers due to the proximity of seed sources, their biological affinity for full sun, the soil types and the conditions that likely prevailed in the abandoned pasture that became the Dayville woodlot.

### Root dynamics

**Figure 4.**
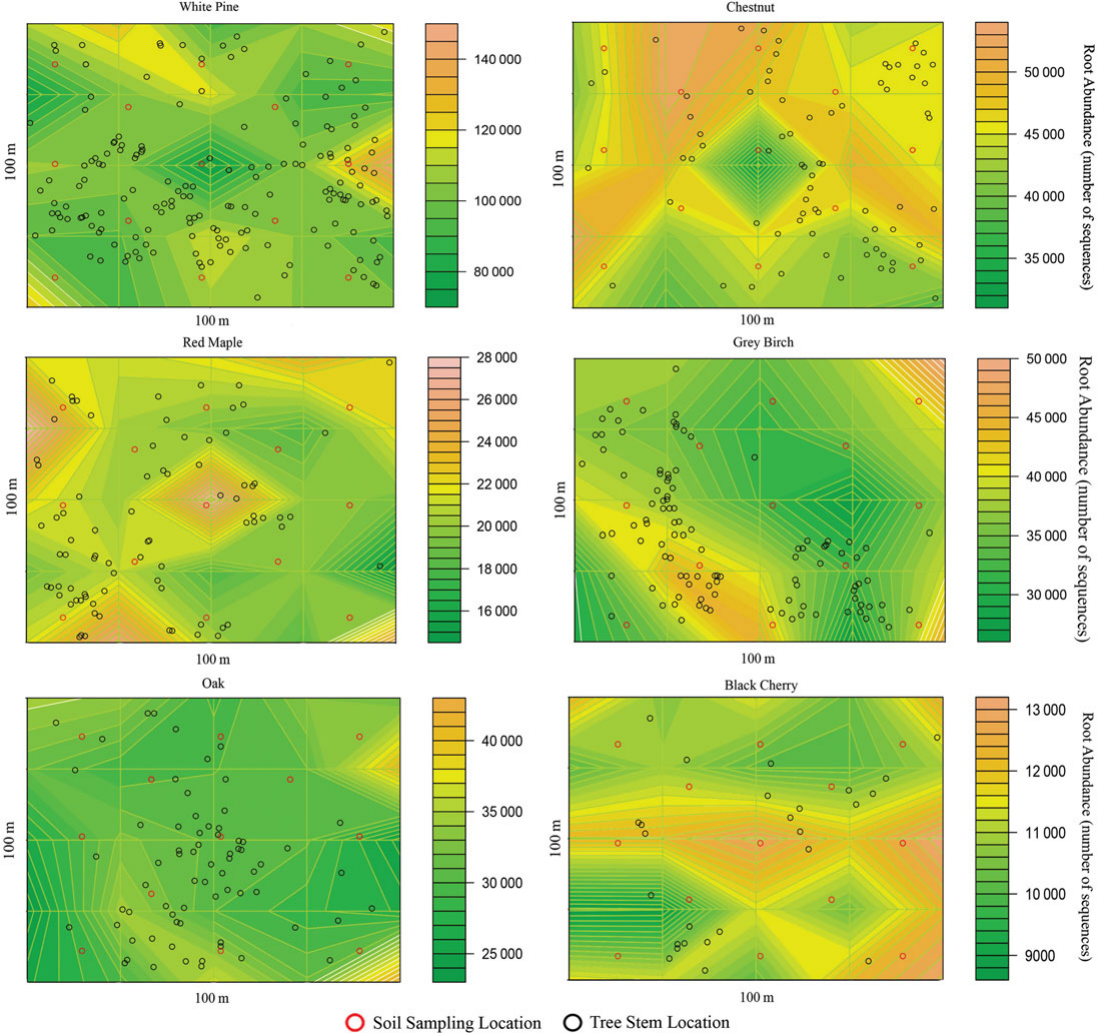
Roots of all genera are distributed throughout the Dayville woodlot, but abundance varies spatially per genus. Warm colours indicate higher root abundance.

**Figure 5.**
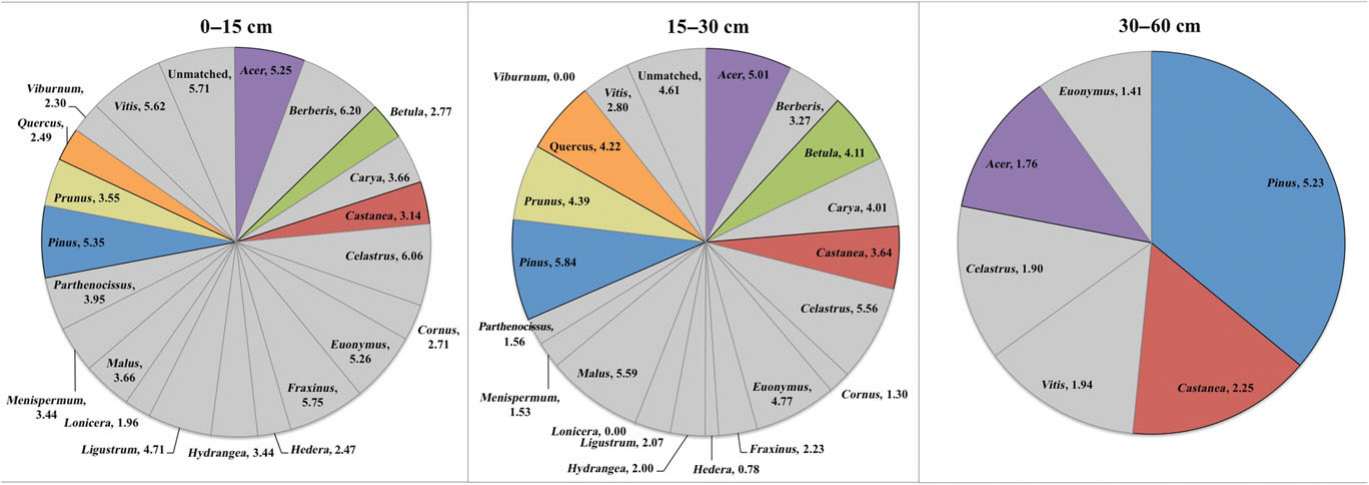
Abundance of roots of all woody genera in the Dayville woodlot at three different depths. Values are the log transformation of the number of sequence reads. Species of interest to this study are highlighted.

Analysis of DNA sequences and inverse modelling showed that the roots of common woody genera were distributed across the forest, and their abundance was variable across the woodlot and at different depths, but the abundance and distribution of roots did not necessarily reflect the abundance and distribution of stems (Fig. 4). Roots of *Castanea* were found throughout the forest but were not associated with microsite or BA (data not shown). Roots of shrub genera were concentrated in the upper 15 cm of the soil, with the exception of *Euonymus*, which was found down to the 30–60 cm range. Trees generally comprised a smaller proportion of the root biomass in the upper 15 cm of soil and a larger proportion below 15 cm (Fig. 5). *Pinus*, *Castanea* and *Acer* made up the majority of roots found at the 30–60 cm depth range. Vine roots from *Celastrus* and *Vitis* occurred in large numbers throughout the depth profile. Large stems of these vines were observed throughout the forest.

### Tree health and vigour

**Figure 6.**
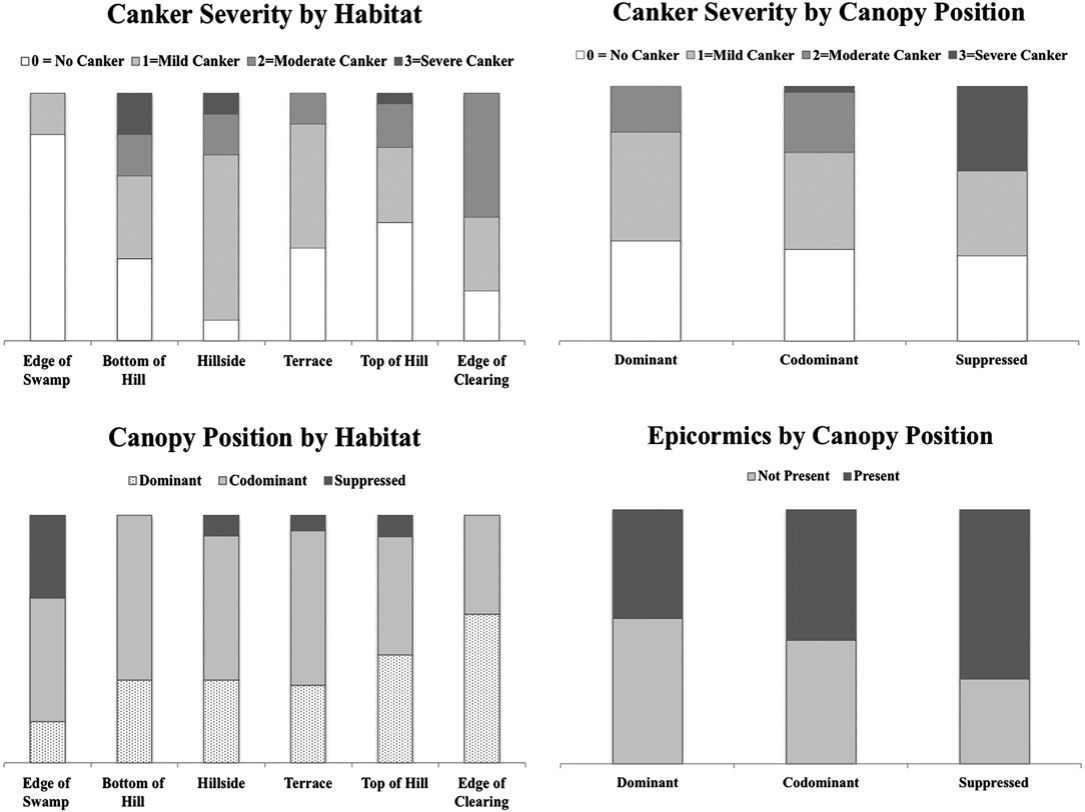
Association of canker severity with habitat and canopy position and canopy position with habitat and presence of epicormic branches for chestnut. Canker severity by habitat: *χ*^2^ = 17.462, df = 10, *P* = 0.0647. Canopy position by habitat: *χ*^2^ = 15.613, df = 15, *P* = 0.482. Canker severity by canopy position: *χ*^2^ = 17.271, df = 9, *P* = 0.0446. Presence of epicormic branches by canopy position: *χ*^2^ = 3.515, df = 3, *P* = 0.3188.

The spatial distribution of chestnut size classes, canopy positions, disease severity and microsites revealed no evident groupings of these attributes. There were no significant associations of attributes except that severe canker was significantly associated with suppressed canopy position (*χ*^2^ = 17.271, df = 9, *P* = 0.0446; Fig. 6). Canker severity was not affected by habitat (*χ*^2^ = 17.462, df = 10, *P* = 0.0647). There were no significant associations between canopy position and habitat (*χ*^2^ = 15.613, df = 15, *P* = 0.482), nor between canopy position and the presence/absence of epicormic branches (*χ*^2^ = 3.515, df = 3, *P* = 0.3188). The power of these tests was limited by small sample sizes: canopy position (dominant = 29, co-dominant = 37, intermediate = 0, suppressed = 6), habitat (edge of swamp = 6, bottom of hill = 6, hillside = 12, terrace = 15, top of hill = 23, edge of clearing = 10), canker severity (no canker = 27, small-healed canker = 28, large-healed canker = 14, severe canker = 3) and epicormic branches (absent = 37, present = 35).

### Dendrochronology

Growth rings indicated that although *C. mollissima* stems varied somewhat in size, they were similar in age; all were around 50 years old, and they comprised a single cohort. The growth curve indicated that the *C. mollissima* trees had grown freely without prolonged periods of suppression or release, and the JOLTS analysis confirms that there were no significant suppression or release events in any of the sampled trees, and therefore no indications of stand-wide disturbance in the forest, at any of the sensitivity levels.

**Figure 7.**
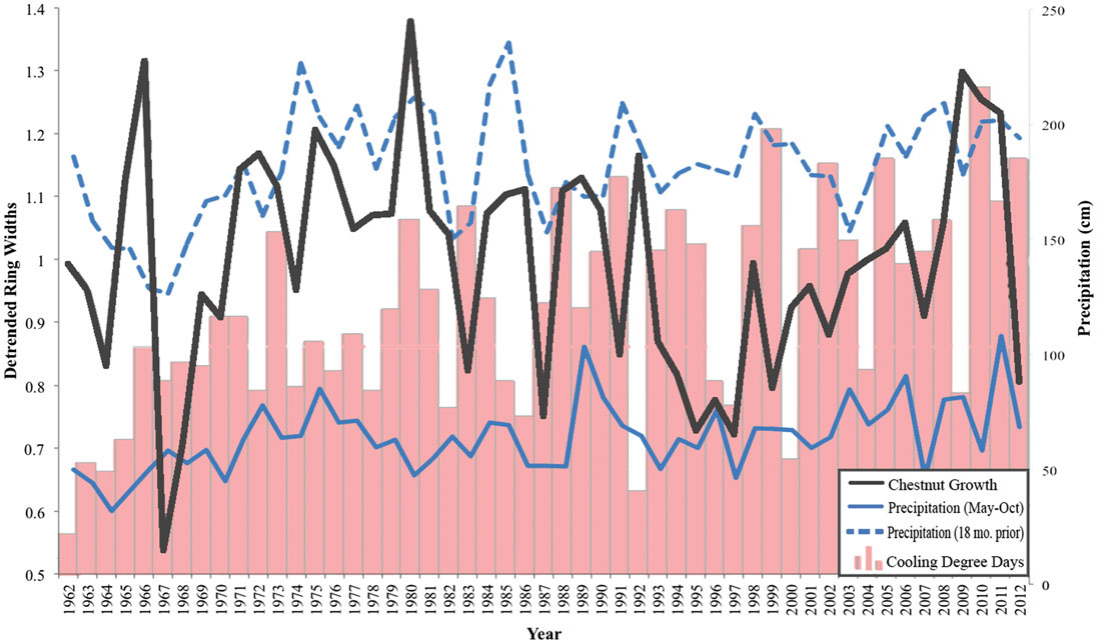
Master chronology of Chinese chestnut growth (detrended ring widths) overlaid on growing-season precipitation, previous 18 months' precipitation and cooling degree days (to show relative heat and light over the years). Multiple regression reveals that growing-season precipitation (*B* = 0.255, *P* = 0.125) and previous 18 months' precipitation (*B* = 0.16, *P* = 0.131) are significantly related to *C. mollissima's* increment growth (*P* = 0.041), but only account for a small part of the variation (*R^2^* = 0.124).

A master chronology of growth increments for *C. mollissima* compared with precipitation and cooling degree days (an indication of growing-season heat) in CT revealed a mixture or significant and non-significant correlations between growth and precipitation windows (Fig. 7). Growth was correlated (at *α* = 0.05) with growing-season precipitation (*P* = 0.042, *R^2^* = 0.081) and the previous 18 months' precipitation (*P* = 0.044, *R^2^* = 0.079), but not with the previous autumn's precipitation (*P* = 0.895, *R^2^* = 0.000), each year's annual precipitation (*P* = 0.325, *R^2^* = 0.019) or with cooling degree days (*P* = 0.885, *R^2^* = 0.000). Mixed linear models revealed that growing-season precipitation (*B* = 0.255, *P* = 0.125) and previous 18 months' precipitation (*B* = 0.16, *P* = 0.131) were the only combination of tested factors significantly correlated with growth (*P* = 0.041, *R^2^* = 0.124).

### Relatedness and parentage

**Figure 8.**
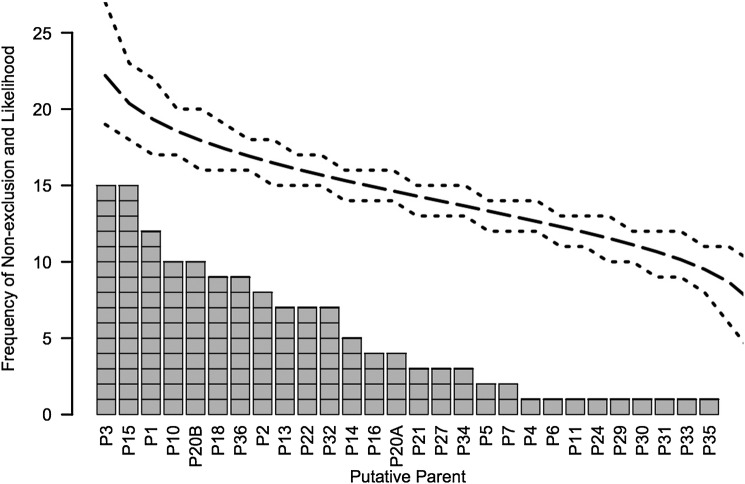
Frequency of chestnut parent contribution to the offspring population. Parents were assigned to offspring based on statistical likelihood or non-exclusion due to matching alleles. Long dashed line is the observed parent randomness, and dotted lines are 95 % confidence envelope for randomness.

We produced a unique microsatellite genotype for each *Castanea* on the site based on 11 loci (Table 2). A PCA of genetic distance showed all trees in both parent and offspring populations to be effectively the same population with high pairwise relatedness among all trees but few fixed alleles in the population (*F*_st_ = 0.005, *F*_is_ = 0.072, *F*_it_ = 0.077). Molecular variance within individuals was 92 %, among individuals was 7 % and among populations (parents versus offspring) was 1 %. Parent and offspring populations shared many alleles, with the exception of three private alleles in the parent population and seven private alleles in the offspring population (Table 3). Most loci were in Hardy–Weinberg equilibrium except CM396, CM800 and CM004 in the parent population and CM396, CM467 and CM800 in the offspring population. The CERVUS analysis of parentage showed that each parent contributed at least one offspring, with some parents contributing to as many as 15 offspring. There was no genetic bottleneck evident, and parental contribution to the offspring population was random (Fig. 8).

**Table 2.** SSR markers used in relatedness analysis. *From Inoue *et al.* (2009). ^†^From Kubisiak *et al.* (2013).

Locus	Primer sequence (5′–3′)	Size (bp)	Repeat	No. of alleles
CM004*	F:ACCACAGAGAGAGCCACACC	189–270	CT	7
	R:TTTCATGAGCACGAAAGCTG			
CM005*	F:AAATAAAACCCCTCATCAACACA	156–182	CT	8
	R:GAACTCAAAACCTCAAAACCTCA			
CM007*	F:TAGTCACGCCTCTCCGTCTT	217–299	AG	5
	R:GCCATTTGAGGACTGAGGTT			
CM008*	F:CCCAAAATCAAAGTCTGAGCA	143–159	AC	8
	R:CGTCGACTCTTCTCTATCTCCAA			
CM010*	F:GTTGGAGAGGTCGTCTCACG	231–255	(CT)(GT)	6
	R:ATTGCGAGGAAAAGGAAACA			
CM018*	F:ACAACGATCCCAGACCAAAG	172–224	CT	10
	R:CTAGGCGATCGGAGAGAGAC			
CM396^†^	F:AACTCCCACCACTCACATCC	181–207	CACACC	6
	R:GTTTCTTTTTCGGACCATCCAGAACTC			
CM467^†^	F:CCCATGCCTACTACATTACAAA	189–199	TCG	3
	R:GTTTCTTGTGGCCGATGGTGTAGATTT			
CM800^†^	F:TTATGGCAACCCTCCTGTTT	140–154	TC	7
	R:GTTTCTTCTGAAATGATCGATGCTGCT			
CM883^†^	F:CAGCATCAGCACTCGTTCA	204–218	AGC	5
	R:GTTTCTTGGGATTGAGAGGATGAAGCA			
CM945^†^	F:AGTGTGAGTGGGGAAGATGG	215–249	GAG	11
	R:GTTTCTTTTtGGCTTCACTGCCAAAC

**Table 3.** Allele information, heterozygosity and tests for the Hardy–Weinberg equilibrium for each locus in each population.^a^Number of alleles observed in the population. ^b^Observed heterozygosity. ^c^Expected heterozygosity. ^d^Number of private alleles per locus, with frequency in parentheses. ^e^Proportion of loci with missing data, averaged over individuals. ^f^Hardy–Weinburg test includes degrees of freedom (df), Chi-square value, *P*-value and significance: ns, not significant; *P < 0.05, **P < 0.01, ***P < 0.001.

Population	Locus name	Na^a^	Ho^b^	He^c^	PA^d^ (frequency)	Missing^e^	Tests for Hardy–Weinburg equilibrium^f^			
							df	*χ*^2^	*P*-value	Significance
Orchard parents (*N* = 28)	CM005	8.000	0.929	0.783	0	0	28	34.677	0.179	ns
	CM008	7.000	0.893	0.798	0	0	21	19.617	0.546	ns
	CM010	6.000	0.667	0.652	1 (0.019)	0	15	8.031	0.923	ns
	CM018	10.00	0.815	0.762	1 (0.019)	0	45	33.842	0.888	ns
	CM396	5.000	1.000	0.678	0	0	10	29.596	0.001	***
	CM467	3.000	0.852	0.647	0	0	3	7.078	0.069	ns
	CM800	7.000	0.741	0.765	0	0	21	36.486	0.019	*
	CM004	5.000	0.550	0.679	0	0.04	10	19.960	0.030	*
	CM007	5.000	0.565	0.723	1 (0.022)	0.07	10	10.517	0.396	ns
	CM883	4.000	0.481	0.488	0	0.04	6	4.150	0.656	ns
	CM945	9.000	0.818	0.801	0	0.07	36	44.043	0.168	ns
Forest offspring (*N* = 72)	CM005	8.000	0.881	0.790	0	0	28	31.201	0.308	ns
	CM008	8.000	0.819	0.776	1 (0.007)	0	28	31.012	0.317	ns
	CM010	5.000	0.754	0.683	0	0	10	13.208	0.212	ns
	CM018	9.000	0.859	0.777	0	0	36	32.050	0.657	ns
	CM396	6.000	0.871	0.635	1 (0.036)	0	15	40.196	0.000	***
	CM467	3.000	0.886	0.666	0	0	3	15.943	0.001	**
	CM800	7.000	0.761	0.750	0	0	21	32.702	0.050	*
	CM004	7.000	0.577	0.577	2 (0.024)	0.04	21	19.632	0.545	ns
	CM007	4.000	0.473	0.604	0	0.014	6	12.467	0.052	ns
	CM883	5.000	0.455	0.520	1 (0.009)	0.027	10	8.911	0.541	ns
	CM945	11.00	0.892	0.819	2 (0.019)	0.014	55	51.033	0.627	ns

## Discussion

*Castanea mollissima* is not native to North America, and while it has been cultivated and grown commercially, it is rarely found integrated into North American forests (Schlarbaum *et al.* 1994). In the Dayville woodlot, however, 50-year-old *C. mollissima* trees were numerous, co-dominant in the canopy and randomly distributed alongside native North American species. No native *C. dentata* had been found in the area for at least 100 years. The species composition, size-class distributions and historical records of the land and forest suggested that it developed through natural colonization and succession and had not been directly managed by humans (Figs 1–3). The presence and age of the trees, along with the mix of significant and non-significant relationships between precipitation, temperature and growth, indicated that the climate and soil were sufficient for *C. mollissima* to establish and to maintain their canopy position as the forest structure developed, and the growth increment data showed little competition pressure or periods of adversity in the past (Fig. 7).

Ecologically, the forest-grown *C. mollissima* resemble *C. dentata*, with several analogues to the development of a disjunct stand of *C. dentata* growing outside of its range in West Salem, WI (Paillet and Rutter 1989). In WI, *C. dentata* was functionally an exotic species that colonized land with shallow, sandy soil of low pH. As at Dayville, the West Salem site had been released from grazing, and pioneer trees grew there under open conditions without competition from other large trees, becoming dominant or co-dominant in the canopy alongside *Quercus*, *Carya*, *Betula* spp. and others (Paillet and Rutter 1989). Despite the fact that the founding population in West Salem was small and closely related (like the *C. mollissima* in our study), the trees in WI did not show any detrimental effects of inbreeding and exhibited heterozygous advantage (Pierson *et al.* 2007). The West Salem *C. dentata* comprised ∼25 % of the BA in the forest, similar to the almost 20 % of *C. mollissima* found at Dayville; and like the *C. mollissima* at Dayville, the growth forms of the *C. dentata* in WI ranged from multi-stemmed and shrub-like to single-stem erect trees (Paillet and Rutter 1989). Tree-ring analyses of the *C. dentata* in WI also showed no direct relationship between tree growth and precipitation or heat (Paillet and Rutter 1989; McEwan *et al.* 2006). In contrast, however, the *C. dentata* population has been dying back and diminishing due to the introduction of chestnut blight (Paillet and Rutter 1989; McEwan *et al.* 2006; Gilland *et al.* 2012), while the *C. mollissima* in CT have continued to grow in the forest despite the presence of chestnut blight.

### Chestnut blight

Chestnut blight cankers were found on almost all of the *C. mollissima* trees, but there was no evidence of mortality caused by blight, similar to previous observations in the parent orchard (Jaynes 1965). Blight severity did not appear to be correlated with differences in site characteristics or location (Fig. 6). There was no link between blight severity and parentage. Because of the long infestation of chestnut blight in the area, it seems that all surviving *C. mollissima* parent trees were capable of producing blight-tolerant offspring. Canopy suppression was the only negative impact observed on the most severely blighted offspring (Fig. 6). The suppressed *C. mollissima* individuals appeared to have become so in recent years, and in addition to canopy suppression they also suffered from stressors such as wind damage and vine competition. In contrast, other *Castanea* species such as *C. dentata* and *C. sativa* (European chestnut) either die or are at a severe competitive disadvantage when exposed to chestnut blight (Paillet 2002; Zlatanov *et al.* 2013). This means that *C. mollissima* can survive and compete in the forest without complete blight resistance, but trees in the lower end of the resistance spectrum are expected to grow more poorly. Based on the observations of wild *C. mollissima* in China (Steiner *et al.* 2009) and suppressed *C. dentata* stump sprouts in the northeastern USA (Paillet 1984), it appears that blight severity causes canopy suppression more than canopy suppression worsens blight symptoms. There appears to be genetic variation in blight resistance within pure *C. mollissima* populations (Steiner *et al.* 2009), indicating that not all genes for blight resistance are fixed in the species. The presence of blight-susceptible *C. mollissima* individuals requires an explanation as to why natural selection in China has not purged blight-susceptible alleles from the population. Current hypothetical models for the genetics of blight resistance (Burnham 1981; Burnham *et al.* 1986; Hebard 1994, 2006) do not account for the variability in blight resistance in *C. mollissima* over evolutionary time.

### Genetic relatedness and heterozygosity

All *C. mollissima* trees in the Dayville woodlot, parents and offspring, were closely related and effectively a single breeding population. Mating and parentage appeared random, but because there were 67 orchard trees planted originally, and because all forest *C. mollissima* are the offspring of at least one of the 28 surviving orchard trees, it is likely that selection pressure has already worked against the non-viable parents and offspring, leaving no trace of the unfit and poorly adapted trees. Of the SSRs used for determining relatedness, CM396 had unusually high heterozygosity and was significantly out of Hardy–Weinberg equilibrium in both the parents and the offspring. CM800 and CM467 also had unusually high heterozygosity, especially in the forest offspring population (Table 3). These markers are located within the quantitative trait locus that confers resistance to the root pathogens in the genus *Phytophthora* (Tatiana Zhebentyayeva, Clemson University, personal communication), which are known to be detrimental and/or lethal to *Castanea* species, especially *C. dentata* (Rhoades *et al.* 2003). It is possible the deviations from allele frequency expectations that we observed at these loci are the consequence of selection favouring the heterozygous state.

### Invasive ecology of *C. mollissima* and co-occurring species

The scarcity of other unmanaged populations of *C. mollissima* throughout eastern North America calls to question why the species has not been as successful elsewhere. It has been suggested that *C. mollissima* cannot compete with native North American forest species because of its short mature canopy height and spreading growth habit (Griffin 2000; Fei *et al.* 2012). Other factors potentially limiting the spread of *C. mollissima* into North American forests include predation of seeds and seedling herbivory by deer, as well as direct competition from invasive understorey plant species. At the Dayville site, shallow (average 30 cm deep) sandy soils on top of granite bedrock limited forest canopy height to ∼18–20 m, and *C. mollissima* trees were able to grow to that height and maintain a competitive position in the canopy. Additionally, it is likely that low predation pressure from a relatively small deer population in CT prior to 1965 allowed more seeds to be dispersed and hoarded by squirrels (Jaynes 1965). We found that *C. mollissima* were producing nuts in this forest, as evidenced by chestnut burs found on the ground under offspring trees, but there appeared to be no tree seedlings, other than several *Fraxinus* seedlings, under 5 cm DBH. The understorey was dominated by non-native shrubs such as *Lonicera*, *Berberis*, *E. alatus* and *Ligustrum* that could outcompete tree seedlings and shelter seed predators such as rabbits and small rodents. The root analysis revealed high abundance of, and therefore high below-ground competition from exotic invasive species such as *E. alatus*, *Berberis* spp. and *Celastrus orbiculatus* (Fig. 5). It follows that natural reproduction of any *Castanea* species, including blight-resistant hybrids, will face the same difficulties as *C. mollissima* and many North American forest natives (Richardson *et al.* 2011; Nuttle *et al.* 2013).

There are concerns that future *C. mollissima* × *C. dentata* hybrids could become invasive and displace native hardwoods (Jacobs 2007; Jacobs *et al.* 2013); however, evidence from the Dayville woodlot and the basic biology of *Castanea* spp. make this unlikely. Problematic invasive woody species in North America, such as *Ailanthis altissima*, *Lonicera* spp., *Rhamnus cathartica*, *Pyrus calleryana* and *Elaeagnus umbellata*, are aggressive partly because they have small seeds that are dispersed by wind or passed through the guts of animals, giving them a wide dispersal range, and they tend to be broadly adapted to varying soil and light conditions (Binggeli 1996; Rejmánek 1996; Guo *et al.* 2000; Zheng *et al.* 2004). *Castanea* seeds are not wind dispersed and are killed when consumed by animals, rather than passing through guts intact. Predation pressure impedes, not enables, their dispersal until the predator population is satiated. Additionally, all *Castanea* spp. are similar in terms of their site requirements and climatic limits (Fitzsimmons 2006; Hunt *et al.* 2009; Fei *et al.* 2012), which means that they are broadly adapted climatically, but narrowly adapted edaphically (McCament and McCarthy 2005; Mellano *et al.* 2012). As such, it is likely that *Castanea* seedlings only exhibit growth advantage over other hardwood genera such as *Quercus* and *Prunus* under optimal soil conditions. This probably explains the discrepancy in the studies of Paillet and Rutter (1989), Jacobs and Severeid (2004), McEwan *et al.* (2006) and Jacobs *et al.* (2009), where *Castanea* was found to have superior growth over other genera, and Clark *et al.* (2012), Griscom and Griscom (2012), and Gauthier *et al.* (2013) where *Castanea* did not outperform other genera. Finally, *Castanea* species are nutritious to wildlife and to humans (McCarthy and Meredith 1988; Senter *et al.* 1994; Paillet 2006; Burke 2013), provide complex habitat structure in forest communities (Zlatanov *et al.* 2013) and mast even under extreme weather conditions such as drought (Gilland *et al.* 2012); therefore, they could be beneficial to future natural and anthropogenic landscapes. Synthesis from the literature and Dayville *C. mollissima* observations predict that *Castanea* hybrids can proliferate in certain areas of North America and confer ecological benefits while not completely displacing other desirable genera such as *Quercus* and *Prunus*.

## Conclusions

The seeming ease with which *C. mollissima* established in the Dayville woodlot begs for an explanation as to why similar stands of *C. mollissima* are rare elsewhere in North America. Likely explanations are that a rare time window of low seed and seedling predation allowed seedlings to establish, and the shallow soil depth at this particular site limited the growth of the native forest canopy to a height attainable by the characteristically short-statured *C. mollissima*. Hybrids may need the genetic potential to grow taller than *C. mollissima* on more productive sites if they are to persist. However, most of the *C. mollissima* germplasm in the USA was selected for nut production and so the full range of its growth potential in forest environments has yet to be explored. The formation of this stand of *C. mollissima* and the disjunct stand of *C. dentata* in WI indicates that *Castanea* species and their hybrids may play a valuable ecological role as pioneer species in the reforestation and reclamation of degraded lands such as fallow farm fields. Short-statured, *C. mollissima*-like hybrids may be desirable components of highly disturbed, anthropogenic environments, conferring more economic and ecological benefits than other exotics that have become invasive. These results imply that introduced tree species in North America could be assessed not only by their level of threat to native species (Parker *et al.* 1999) but also by their potential positive impacts on North American ecosystems and their usefulness in programmes to breed certain resilient traits into native North American species.

## Sources of Funding

Our work was funded by a Fred M. van Eck scholarship from the Hardwood Tree Improvement and Regeneration Center at Purdue University, West Lafayette, IN, USA.

## Contributions by the Authors

A.C.M. performed most of the field measurements, laboratory analyses, data analyses and table/figure creation, and wrote most of the manuscript. K.E.W. provided guidance on genetic analyses and interpretations and helped write the manuscript. S.L.A. provided access to the study site, background information for the study and helped write the manuscript. D.F.J. contributed to experimental design and logistics, and helped write the manuscript.

## Conflicts of Interest Statement

None declared.
